# Mitochondria as an Integrative Hub of Cellular Homeostasis and Stress Response

**DOI:** 10.3390/ijms27093871

**Published:** 2026-04-27

**Authors:** Valentina Mihaylova, Eleonora Kovacheva, Maria Gevezova, Victoria Sarafian, Maria Kazakova

**Affiliations:** 1 Department of Medical Biology, Medical University of Plovdiv, 15A Vasil Aprilov Blvd., 4002 Plovdiv, Bulgaria; eleonora.kovacheva@mu-plovdiv.bg (E.K.); maria.gevezova@mu-plovdiv.bg (M.G.); victoria.sarafian@mu-plovdiv.bg (V.S.); maria.kazakova@mu-plovdiv.bg (M.K.); 2 Research Institute, Medical University of Plovdiv, 4002 Plovdiv, Bulgaria

**Keywords:** mitochondrial function, rheumatoid arthritis, ischemic stroke, autism spectrum disorder

## Abstract

Mitochondria are increasingly recognized as multifunctional organelles that integrate metabolic, redox, immune, and cell fate signaling, thereby maintaining cellular and tissue homeostasis under physiological conditions. Beyond their classical role in ATP production, mitochondria act as central regulatory hubs coordinating adaptive responses to metabolic demands and environmental stress. These functions are sustained through tightly regulated quality control mechanisms, including mitochondrial biogenesis, dynamic fusion–fission remodeling, redox signaling, and selective removal of damaged organelles via mitophagy. Disruption of these processes compromises cellular resilience and contributes to disease initiation and progression. This review summarizes and critically evaluates current evidence on mitochondrial function in health and its dysregulation in pathological conditions, with a particular focus on rheumatoid arthritis (RA), ischemic stroke (IS), and autism spectrum disorder (ASD). Despite their distinct clinical manifestations, these disorders share convergent mitochondrial abnormalities, including metabolic reprogramming toward glycolysis, excessive or persistent reactive oxygen species production, impaired mitophagy, mitochondrial DNA-driven innate immune activation, and hypoxia-related stress. In RA, mitochondrial dysfunction sustains chronic inflammation and joint destruction; in IS, acute mitochondrial failure and reperfusion-associated oxidative stress drive neuronal injury; and in ASD, mitochondrial metabolic inflexibility and defective quality control contribute to chronic low-grade inflammation and neurodevelopmental vulnerability. A variety of methods for the assessment of mitochondrial function are available to study these pathological conditions. Collectively, these findings position mitochondrial dysfunction as a unifying pathogenic mechanism linking inflammatory, neurodegenerative, and neurodevelopmental processes. Targeting mitochondrial metabolism, redox balance, and quality control pathways therefore represents a promising cross-disease therapeutic strategy.

## 1. Mitochondrial Function in Physiological Conditions

Mitochondria are double-membrane organelles found in the cytoplasm of all eukaryotic cells. Their primary function is to provide an energy source for cellular processes [1]. The two membranes are composed of a phospholipid bilayer, with the outer membrane serving to separate the cytosol from the matrix, while the inner membrane is folded to increase the surface area for the protein complexes of the electron transport chain (ETC). The resulting folds are called cristae [2]. The permeability of the two membranes differs markedly. The outer membrane is more permeable and contains hydrophilic pores that allow the passage of metabolites and ions, whereas the inner membrane is characterized by protein translocases that actively transport molecules [3].

The process of cellular respiration is the most fundamental function of living organisms. It occurs through the electron transport chain (ETC), which is located on the inner mitochondrial membrane [4]. Through oxidative phosphorylation (OXPHOS) in the ETC, adenosine triphosphate (ATP) is generated, which is essential for the survival and proper functioning of the cell [5]. ATP also acts as an important extracellular signaling molecule. Extracellular ATP promotes neutrophil chemotaxis and the release of IL-8 and elastase from neutrophils. It stimulates phagocytosis of exogenous pathogens, induces neutrophil degranulation, and increases reactive oxygen species (ROS) production [6]. Another mechanism linking ATP to inflammation is through the activation of the NLRP3 inflammasome [7]. Binding of ATP to the P2X7 receptor (P2X7R) on the surface of immune cells opens an ion channel and triggers rapid efflux of potassium from the cytosol, leading to inflammasome activation and caspase-1 cleavage [8].

**Figure 1:**
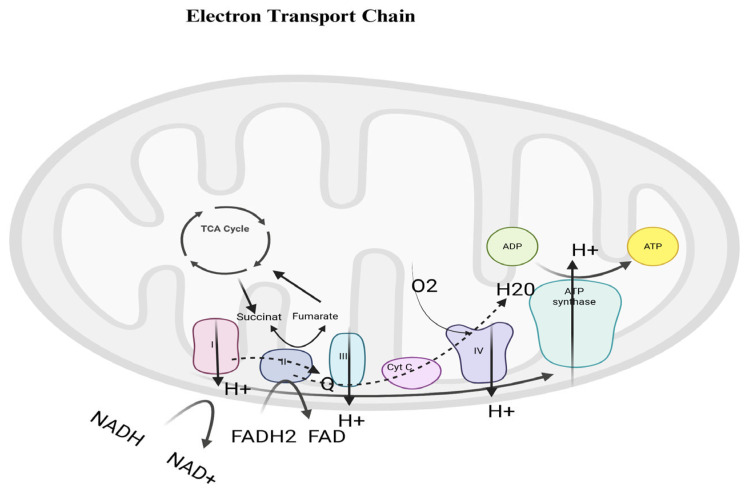
NADH/NAD^+^—donates electrons to Complex I and is oxidized to NAD^+^, FADH_2_/FAD—donates electrons to Complex II and is oxidized to FAD, Complex I (NADH dehydrogenase)—transfers electrons and pumps H^+^ into the intermembrane space, Complex II (succinate dehydrogenase)—transfers electrons (does not pump H^+^), Coenzyme Q (ubiquinone)—carries electrons from Complex I/II to Complex III, Complex III (cytochrome bc1 complex)—transfers electrons and pumps H^+^, Cytochrome c (Cyt C)—transfers electrons to Complex IV, Complex IV (cytochrome c oxidase)—transfers electrons to O_2_ and forms H_2_O, O_2_—final electron acceptor, H^+^ (protons)—build a proton gradient in the intermembrane space, ATP synthase—uses the proton gradient to produce ATP, ADP + Pi → ATP—process of ATP formation, TCA cycle (Krebs cycle)—produces NADH and FADH_2_.

The OXPHOS system is composed of five enzyme complexes and two electron carriers [9]. Two electrons from NADH are transferred to Complex I (NADH dehydrogenase), resulting in the pumping of four hydrogen ions (H^+^) across the inner mitochondrial membrane. NADH is oxidized to NAD^+^ and recycled back to the Krebs cycle. FADH_2_ is generated during the oxidation of succinate to fumarate within Complex II and transfers electrons to ubiquinone without proton pumping. Reduced ubiquinone is oxidized to ubiquinol, and electrons are transferred to another carrier, cytochrome c, which delivers them to Complex IV (cytochrome c oxidase), where an additional two H^+^ ions are pumped. Finally, the electrons reach an oxygen molecule, which is split into atoms. These atoms then combine with H^+^ ions to form two molecules of water. The fifth complex, ATP synthase, returns the pumped H^+^ ions from the ETC back into the matrix. The energy released by the proton flow is used to phosphorylate adenosine diphosphate (ADP) to ATP. Overall, oxidative phosphorylation contributes to the generation of approximately 30–32 ATP molecules per glucose molecule under optimal conditions, based on the combined oxidation of NADH and FADH_2_, although the exact yield may vary depending on cellular and physiological conditions [10,11]. The process is illustrated in Figure 1.

Traditionally viewed as the primary site of cellular energy production, mitochondria are now widely recognized as multifunctional organelles that integrate metabolic, redox, immune, and cell fate signaling. Rather than acting as passive “powerhouses,” mitochondria function as central regulatory hubs that coordinate cellular responses to physiological demands and environmental cues, thereby maintaining cellular and tissue homeostasis [12,13]. Disruption of this integrative role has profound consequences for tissue function and contributes to the initiation and progression of numerous diseases [14].

Under physiological conditions, mitochondrial function is sustained through a highly coordinated network of quality control mechanisms, including mitochondrial biogenesis, dynamic remodeling of the mitochondrial network, and selective removal of damaged organelles via mitophagy. These processes enable cells to fine-tune mitochondrial mass, morphology, and bioenergetic capacity in response to fluctuating metabolic requirements and signaling inputs [15]. Mitochondrial biogenesis is governed by tightly regulated nuclear–mitochondrial crosstalk involving transcriptional coactivators and transcription factors such as PGC-1α, NRF1/2, and TFAM, which collectively ensure balanced expression of nuclear- and mitochondrial-encoded components of the oxidative phosphorylation machinery [16].

Mitochondrial dynamics, characterized by continuous cycles of fusion and fission, are essential for maintaining mitochondrial integrity and functional adaptability. Fusion facilitates the complementation of mitochondrial contents and supports efficient energy production, whereas fission is required for mitochondrial redistribution, inheritance during cell division, and segregation of dysfunctional mitochondria for degradation. Even subtle disturbances in this dynamic equilibrium can impair metabolic efficiency and cellular resilience, underscoring the importance of mitochondrial morphology in biological homeostasis [17].

A central aspect of mitochondrial physiology is the regulation of programmed cell death pathways, including apoptosis and necroptosis. Through control of mitochondrial outer membrane permeabilization, release of cytochrome C, and subsequent caspase activation, mitochondria act as decisive regulators of cell fate [18]. Importantly, mitochondrial signaling pathways involved in cell death are closely intertwined with adaptive stress responses, allowing mitochondria to function as molecular switches that determine whether cells survive transient challenges or undergo elimination.

While mitochondria are classically known for their role in energy production through oxidative phosphorylation, emerging evidence highlights their function as critical signaling organelles. Beyond ATP generation, mitochondria communicate with the nucleus, peroxisomes, and other cellular compartments to regulate metabolic adaptation, apoptosis, and cellular differentiation. This signaling role allows mitochondria to influence processes such as cancer initiation and progression, immune response, and intercellular communication, independent of their energetic output [19]. Understanding mitochondria as signaling hubs provides a broader perspective on their involvement in disease, complementing their traditional bioenergetic characterization.

Mitochondria are an important intracellular source of superoxide and other ROS, while also representing a major redox-sensitive target of oxidative damage. At controlled physiological levels, mitochondrial ROS serve as essential signaling molecules that regulate gene expression, immune activation, and cellular adaptation to metabolic and environmental stress [20]. Redox signaling originating from mitochondria modulates pathways involved in hypoxic responses, metabolic reprogramming, and stress tolerance, thereby contributing to cellular homeostasis [21]. In contrast, mitochondrial dysfunction results in excessive ROS production, leading to oxidative damage of lipids, proteins, and mitochondrial DNA (mtDNA). All these events determine the link between bioenergetic failure and chronic inflammatory signaling [22].

Beyond their metabolic and redox functions, mitochondria play a pivotal role in innate immune regulation. Reflecting their evolutionary bacterial origin, mitochondrial constituents such as mtDNA, cardiolipin, and N-formyl peptides can act as danger-associated molecular patterns (DAMPs). When released or exposed following mitochondrial stress or damage, these molecules activate innate immune sensors including TLR9, the cGAS–STING pathway, and the NLRP3 inflammasome, thereby converting mitochondrial perturbations into sterile inflammatory responses [23,24]. Under physiological conditions, however, controlled mitochondrial signaling contributes to immune surveillance and the fine-tuning of inflammatory responses, highlighting the dual role of mitochondria as both initiators and modulators of immunity [25].

Importantly, mitochondria serve as key mediators of the crosstalk between cellular metabolism, inflammation, and tissue-specific functions, particularly in energy-demanding systems such as the immune and nervous systems [26]. In immune cells, mitochondrial metabolic state influences differentiation, effector functions, and memory formation. In neurons, mitochondrial positioning, bioenergetic output, and calcium buffering are critical for synaptic transmission, axonal transport, and neuronal plasticity [27]. Consequently, disturbances in mitochondrial dynamics, metabolic flexibility, or quality control mechanisms exert effects that extend far beyond individual cells, impacting tissue integrity and organismal physiology.

## 2. Mitochondrial Dysfunction in Inflammation and Neurodegenerative Processes

Under pathological conditions, excessive mitochondrial damage or bioenergetic failure can shift regulated cell death toward uncontrolled necrosis, thereby amplifying tissue injury and inflammation [28].

**Figure 2:**
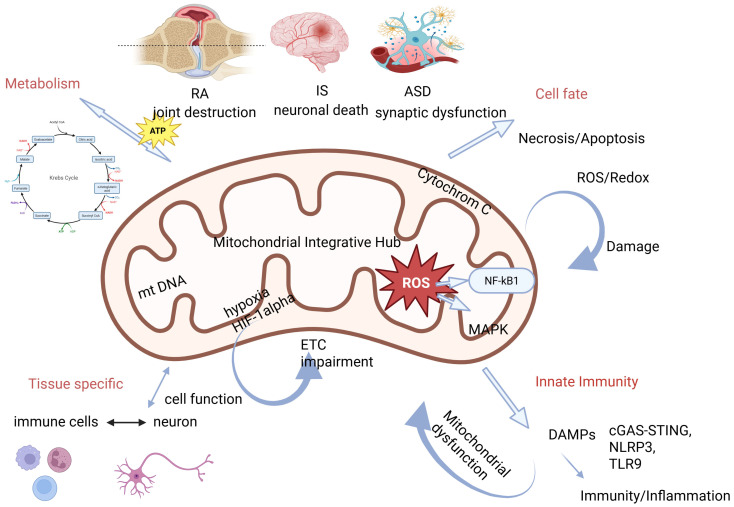
Mitochondria as a central mechanistic hub integrating metabolic reprogramming, redox imbalance, innate immune activation, and defective quality control across rheumatoid arthritis (RA), ischemic stroke (IS), and autism spectrum disorder (ASD). Mitochondrial dysfunction is characterized by impaired oxidative phosphorylation, increased ROS production, release of mitochondrial DNA, defective mitophagy, and metabolic shifts toward glycolysis. These alterations activate key signaling pathways, including NF-κB, MAPK, and cGAS–STING, and are further modulated by hypoxia-induced HIF-1α signaling. Disease-specific outcomes arise from shared core mechanisms, while feedback loops between oxidative stress, inflammation, and mitochondrial damage sustain pathology.

Despite distinct clinical manifestations, ischemic stroke (IS), rheumatoid arthritis (RA), and autism spectrum disorder (ASD) share a convergent pathophysiological feature: failure of mitochondrial integration across metabolic, redox, immune, and signaling networks. This shared mitochondrial vulnerability provides a unifying framework for understanding how diverse disease phenotypes emerge from common cellular stress responses [29]. Figure 2 represents pathophysiological mechanisms and triggers at the mitochondrial level and their association with RA, IS and ASD.

## 3. Assessment of Mitochondrial Function: An Overview of Experimental Approaches

One of the most widely used approaches to evaluate mitochondrial function is the measurement of oxygen consumption, which reflects the activity of the electron transport chain and oxidative phosphorylation. High-resolution respirometry allows detailed characterization of mitochondrial respiration in isolated mitochondria, permeabilized cells, or intact cells. Parameters such as basal respiration, maximal respiratory capacity, spare respiratory capacity, and coupling efficiency provide insight into mitochondrial adaptability and bioenergetic health. These measurements are considered a gold standard for functional mitochondrial assessment in both basic and translational research [30].

The mitochondrial membrane potential (ΔΨm) is a critical biophysical parameter generated by proton pumping across the inner mitochondrial membrane. ΔΨm is essential for ATP synthesis, metabolite transport, and mitochondrial protein import. It is commonly assessed using potential-sensitive fluorescent dyes such as JC-1, TMRE, or TMRM. Changes in ΔΨm are often among the earliest indicators of mitochondrial dysfunction and are closely linked to apoptotic signaling. However, interpretation of fluorescence-based measurements requires careful control, as dye concentration, mitochondrial mass, and cellular context can influence the signal [31]. Another key indicator of mitochondrial function is ATP production, which represents the ultimate energetic output of oxidative phosphorylation. ATP levels can be quantified using bioluminescent luciferase-based assays or genetically encoded ATP sensors, enabling measurements in bulk samples or at the single-cell level. While ATP quantification provides a direct measure of cellular energy status, it does not by itself identify the specific site or mechanism of mitochondrial impairment and therefore is often combined with respiratory measurements [32].

Mitochondria are also a major source of ROS, generated as by-products of electron transport. Controlled ROS production plays signaling roles, whereas excessive ROS generation contributes to oxidative stress and cellular damage. Mitochondrial ROS can be measured using targeted fluorescent probes such as MitoSOX, as well as biochemical and spectrophotometric assays. Despite their widespread use, ROS measurements remain technically challenging due to probe specificity and the highly reactive nature of these species [33].

In addition to functional readouts, biochemical assays of individual respiratory chain complexes provide mechanistic insight into mitochondrial defects. Spectrophotometric measurements of complexes I–IV activity are frequently used in studies of mitochondrial diseases and toxicology. These assays enable localization of defects within the electron transport chain but are typically performed under in vitro conditions and therefore do not fully reflect mitochondrial dynamics in living cells [34].

Finally, mitochondrial mass, morphology, and dynamics are increasingly recognized as integral components of mitochondrial function. Changes in mitochondrial size, network connectivity, and the balance between fission and fusion are closely linked to metabolic adaptation and stress responses. These parameters are commonly assessed using fluorescence microscopy, electron microscopy, and analysis of proteins involved in mitochondrial dynamics. Fluorescent-labeled antibodies are used to localize proteins within the electron chain. While primarily structural, these measurements provide important context for interpreting functional data [35].

The major experimental approaches currently used to assess mitochondrial function, together with their key advantages and limitations, are summarized in Table 1.

Each method has inherent limitations that should be considered when interpreting mitochondrial function.

Mitochondrial dysfunction in RA, IS, and ASD is not merely a secondary epiphenomenon but represents a measurable and potentially targetable biological axis. In RA, assessment of mitochondrial ROS production, mtDNA levels, and mitophagy efficiency may serve as biomarkers of inflammatory burden and therapeutic response [36]. In IS, early detection of mitochondrial impairment could refine prognostic stratification and guide the use of mitochondria-targeted neuroprotective strategies during reperfusion [37]. In ASD, identification of mitochondrial metabolic signatures may define a biologically distinct subgroup of patients who could benefit from metabolic or redox-modulating interventions. Thus, integrating mitochondrial profiling into clinical research may bridge mechanistic insights with personalized therapeutic strategies across inflammatory, ischemic, and neurodevelopmental disorders [38].

## 4. Mitochondrial Dysfunction in RA

RA is a chronic autoimmune inflammatory disease that primarily affects the joints, leading to pain, stiffness, and progressive joint damage. It is a systemic condition that can also involve other organs and tissues, significantly impacting patients’ quality of life. Early diagnosis and appropriate treatment are essential to slow disease progression and prevent long-term disability [39]. In RA, synoviocytes and chondrocytes play a central role in disease pathogenesis and joint destruction. Activated fibroblast-like synoviocytes contribute to chronic synovial inflammation by producing pro-inflammatory cytokines, chemokines, and matrix-degrading enzymes, leading to pannus formation and invasion of cartilage and bone [40]. Chondrocytes, which are responsible for maintaining cartilage homeostasis, undergo phenotypic changes in the inflammatory environment of the rheumatoid joint, resulting in increased production of matrix metalloproteinases and reduced synthesis of extracellular matrix components. The dysregulated interaction between synoviocytes and chondrocytes accelerates cartilage degradation and contributes to the progressive joint damage characteristic of RA [41].

### 4.1. Metabolic Reprogramming of Immune Cells

RA is characterized by profound metabolic adaptations in both innate and adaptive immune cells that support chronic inflammation and tissue destruction [42]. Activated immune cells in RA preferentially rely on aerobic glycolysis rather than on mitochondrial oxidative phosphorylation, despite adequate oxygen availability, resembling the metabolic phenotype of rapidly proliferating cells [43]. This shift enables fast ATP generation and provides biosynthetic intermediates necessary for cytokine production and effector functions [44].

### 4.2. Oxidative Stress, Hypoxia and Inflammation

Macrophages and T cells isolated from RA patients exhibit increased glucose uptake, enhanced glycolytic flux, and reduced mitochondrial respiration, accompanied by altered expression of metabolic regulators such as HIF-1α and mTOR [45]. Importantly, mitochondrial dysfunction is not merely a consequence of immune activation but dynamically reinforces inflammatory signaling through increased ROS production and impaired mitochondrial quality control, thereby sustaining chronic inflammation [46]. Synovial fibroblasts in RA acquire an aggressive, tumor-like phenotype that drives joint destruction through invasive behavior, excessive cytokine secretion, and matrix degradation [47]. Mitochondria-derived ROS play a central role in stabilizing this pathogenic phenotype. Elevated basal ROS levels promote persistent activation of redox-sensitive signaling pathways, including NF-κB and MAPK cascades, leading to resistance to apoptosis and sustained inflammatory gene expression [48].

Oxidative stress further induces epigenetic and metabolic alterations that lock synovial fibroblasts into a hyperinflammatory and invasive state. Thus, in RA, mitochondrial ROS signaling primarily contributes to tissue destruction by maintaining fibroblast aggressiveness rather than inducing acute cytotoxicity [49]. The RA synovium is characterized by chronic hypoxia resulting from increased metabolic demand, vascular dysfunction, and inflammatory infiltration [50]. Hypoxia stabilizes HIF-1α, promotes glycolysis, suppresses mitochondrial respiration, and enhances inflammatory mediator production. This persistent hypoxic stress sustains long-term metabolic and inflammatory adaptations rather than triggering acute mitochondrial collapse, thereby perpetuating joint pathology [51]. In RA, chronic cellular stress, mitochondrial damage, and defective mitophagy facilitate the release of mtDNA into the cytosol and extracellular space [52]. Rather than triggering acute immune responses, mtDNA contributes to the maintenance of chronic inflammatory circuits within the synovial microenvironment. Elevated mtDNA levels in synovial fluid and plasma correlate with disease activity, positioning mtDNA as a link between mitochondrial damage and persistent immune activation [53].

Efficient mitochondrial quality control is essential for immune cell homeostasis. In RA, impaired mitophagy in macrophages and T cells results in the accumulation of dysfunctional, ROS-producing mitochondria, further amplifying inflammatory signaling [54]. Disruption of key regulators such as PINK1 and Parkin enhances inflammasome activation and pro-inflammatory cytokine release, creating a self-perpetuating cycle of mitochondrial damage and inflammation [55]. Importantly, the apparent discrepancy between TNF-α-induced mitophagy and impaired mitochondrial clearance in RA may reflect differences between mitophagy initiation and effective mitophagic flux. While TNF-α can acutely stimulate mitophagy as a compensatory response, chronic inflammatory exposure and persistent oxidative stress may disrupt lysosomal function and impair completion of the mitophagic process [48,50]. Thus, RA is likely characterized by dysregulated rather than by absent mitophagy.

In addition, recent evidence indicates that defective autophagy and extracellular vesicle-mediated release of post-translationally modified proteins contribute to the generation of autoantigens in RA, further linking mitochondrial quality control failure to chronic autoimmune activation [56].

In RA, mitochondrial dysfunction is likely initially secondary to chronic inflammatory and hypoxic stress but subsequently becomes a self-sustaining amplifier of inflammation.

## 5. Mitochondrial Dysfunction in IS

IS is characterized by an abrupt reduction in cerebral blood flow, leading to acute deprivation of oxygen and glucose which results in rapid mitochondrial dysfunction. Because neurons and glial cells are among the most energy-demanding cell types in the human body, even short periods of ischemia profoundly disrupt mitochondrial oxidative phosphorylation, ATP production, and redox homeostasis. As a consequence, mitochondrial failure represents one of the earliest and most decisive events determining the extent of ischemic brain injury [57].

In the early phase of ischemia, brain cells transiently engage adaptive mechanisms aimed at preserving energy balance and limiting oxidative damage. These include metabolic plasticity, antioxidant responses, and activation of mitochondrial quality control pathways. Experimental evidence indicates that ischemic preconditioning exploits this mitochondrial adaptability to confer neuroprotection, highlighting the central role of metabolic reprogramming in ischemic tolerance [58]. However, when ischemia is prolonged or severe, these compensatory mechanisms become insufficient, and mitochondrial damage progresses toward irreversible failure.

### 5.1. Metabolic Reprogramming and Cellular Adaptation

Metabolic reprogramming is a hallmark of the ischemic brain and involves coordinated changes in glycolysis, oxidative phosphorylation, fatty acid metabolism, and amino acid utilization [56]. Astrocytes play a particularly important role in this process due to their high metabolic flexibility and capacity to support neuronal energy demands. Although astrocytes are predominantly glycolytic under physiological conditions, ischemia further amplifies glycolytic flux while impairing mitochondrial oxidative metabolism. Under ischemic and hypoxic conditions, astrocytes rapidly shift toward glycolysis and increase lactate production, thereby providing an alternative energy substrate for neurons and partially compensating for impaired mitochondrial respiration [57].

Astrocyte reactivity encompasses not only metabolic changes but also alterations in gene expression, morphology, and inflammatory signaling. Distinct astrocytic phenotypes, commonly referred to as A1 and A2 states, differentially influence post-stroke neuroinflammation and neural repair [59]. The metabolic status of astrocytes is increasingly recognized as a key determinant of these functional phenotypes. Genetic or acquired impairments in glucose uptake and utilization, such as those associated with disrupted GLUT1 function, compromise astrocytic metabolic support and exacerbate neuronal vulnerability during ischemia [60].

Thus, metabolic reprogramming in IS represents a double-edged adaptation: while initially protective, prolonged reliance on glycolysis and suppression of mitochondrial respiration ultimately contribute to redox imbalance, inflammatory activation, and cellular dysfunction.

### 5.2. Mitochondrial ROS and Oxidative Injury in IS

Mitochondrial dysfunction during ischemia leads to inhibition of the electron transport chain (ETC), resulting in reduced ATP synthesis and increased leakage of electrons that generate ROS. Experimental studies have demonstrated substantial reductions in ETC activity within hours of ischemia, accompanied by marked declines in ATP levels and accumulation of oxidative damage in affected brain regions [61].

The reperfusion phase represents a critical turning point in ischemic injury. Reintroduction of oxygen into metabolically compromised tissue triggers a burst of mitochondrial ROS production, which exacerbates lipid peroxidation, protein oxidation, DNA damage, and blood–brain barrier disruption [62]. While low levels of ROS participate in physiological signaling and vascular regulation, excessive ROS overwhelm antioxidant defenses and drive neuronal death, edema formation, and secondary injury cascades [63].

### 5.3. Mitochondrial Injury and Mitophagy

Mitochondria are the predominant intracellular source of ROS in the ischemic brain. Under sustained oxidative stress, superoxide anions generated within the ETC react with nitric oxide to form peroxynitrite and other highly reactive species that damage mt DNA, proteins, and membrane lipids [64]. This oxidative injury further impairs mitochondrial function, reinforcing a self-propagating cycle of energy failure and oxidative stress.

Mitochondrial injury is a key initiating event in ischemia-associated reperfusion injury and leads to the release of mitochondrial damage-associated molecular patterns (mtDAMPs), including mtDNA [65]. Once released into the cytosol or circulation, mtDNA activates innate immune pathways and serves as both a trigger and a biomarker of ischemic brain injury [66].

Activation of pattern-recognition receptors such as TLRs, the NLRP3 inflammasome, and the cGAS–STING pathway amplifies post-ischemic inflammation by inducing pro-inflammatory cytokine release, interferon signaling, and pyroptotic cell death. These immune responses contribute to secondary neuronal injury and exacerbate tissue damage beyond the initial ischemic core. Importantly, excessive innate immune activation also interferes with mitochondrial recovery, thereby linking immune signaling to prolonged metabolic impairment.

Mitophagy is rapidly induced in ischemic neurons and glial cells as a protective response aimed at removing damaged mitochondria and limiting ROS production. When appropriately regulated, mitophagy supports neuronal survival and preserves mitochondrial network integrity [67]. However, accumulating evidence indicates that the role of mitophagy in IS is highly dependent on context.

Under mild or transient ischemic stress, limited mitochondrial permeability transition activates mitophagy and facilitates metabolic recovery. In contrast, severe or prolonged ischemia triggers excessive mitochondrial damage, overwhelming autophagic capacity and leading to autophagic dysfunction, apoptosis, or necrotic cell death [68]. Thus, the balance between mitochondrial impairment and mitophagy clearance critically determines ischemic outcome.

### 5.4. Hypoxia, Mitochondria, and Inflammation in IS

Hypoxia represents the primary initiating factor linking mitochondrial dysfunction to inflammation in IS. Oxygen deprivation disrupts mitochondrial dynamics, promotes membrane depolarization, and enhances ROS leakage from the ETC, thereby activating inflammatory signaling pathways [69]. In parallel, hypoxia-induced metabolic shifts toward glycolysis alter redox homeostasis and influence immune cell behavior within the ischemic brain.

Inflammation plays a role in all phases of stroke development, from acute injury to post-ischemic recovery [70]. While initially protective, persistent inflammation becomes maladaptive, interfering with mitochondrial repair and prolonging metabolic dysfunction. Reperfusion injury further intensifies this process through ROS-driven inflammatory amplification, creating a feed-forward loop that limits tissue repair and functional restoration [71].

## 6. Mitochondrial Dysfunction in ASD

ASD is increasingly recognized as a complex systemic condition rather than a purely neurodevelopmental disorder. Beyond its core behavioral and cognitive manifestations, ASD is associated with immune dysregulation, metabolic abnormalities, and mitochondrial dysfunction, which together may contribute to chronic low-grade inflammation and altered cellular signaling [72]. Importantly, mitochondrial dysfunction is observed only in a subset of individuals with ASD, with approximately 5% of children affected by classically defined mitochondrial disease, reflecting the heterogeneity of mitochondrial involvement among patients [38]. Mitochondrial dysfunction in ASD can be primary—arising from genetic defects that directly impair mitochondrial ATP production—or secondary, resulting from other metabolic, biochemical, or environmental factors that indirectly compromise mitochondrial function, highlighting diverse mechanisms contributing to energy metabolism abnormalities in this population [73]. Mitochondrial dysfunction can be classified as either primary or secondary, where primary refers to defects in genes directly involved in mitochondrial energy production and secondary indicates impairment due to other metabolic or genetic abnormalities that disrupt mitochondrial ATP production.

### 6.1. Metabolic Reprogramming and Immune Dysfunction

Under physiological conditions, resting immune cells primarily rely on mitochondrial oxidative phosphorylation, whereas activation induces dynamic shifts toward glycolysis and anabolic metabolism [74]. In ASD, this adaptive flexibility appears to be compromised.

Multiple studies report metabolic signatures consistent with mitochondrial inefficiency in a substantial subset of individuals with ASD. These include lactic acidosis, altered pyruvate and amino acid levels, reduced carnitine and ubiquinone availability, and abnormalities in fatty acid metabolism [75]. This predominantly glycolytic profile resembles that of chronically activated immune cells and may favor sustained production of pro-inflammatory mediators.

Alterations in glutamine metabolism, the glutamate–glutamine cycle, and alternative substrate utilization further suggest that mitochondrial metabolic inflexibility contributes to immune dysfunction in ASD [76,77,78,79]. These metabolic abnormalities may limit the ability of immune cells to appropriately resolve inflammatory responses, thereby promoting chronic immune activation [80]. Further support for this concept comes from the theory of the “cell danger response” (CDR), which involves metabolic transformations requiring cellular reprogramming and changes in mitochondrial phenotype to enable progression through three phases: 1—inflammation, 2—proliferation, and 3—differentiation [81], a process that may underlie autism. CDR induces a programmed shift in mitochondrial activity and is necessary for the establishment of an appropriate adaptive immune response and tissue repair [82].

### 6.2. Oxidative Stress, Redox Imbalance and Neuroinflammation

Oxidative stress is widely considered a key link between immune activation and mitochondrial dysfunction in ASD. ROS generated by environmental exposures and activated immune cells can directly damage mitochondrial components, further impairing energy production and metabolic adaptability [83,84].

Glutathione, the major intracellular antioxidant, plays a central role in maintaining redox homeostasis and requires adequate mitochondrial function for its synthesis. Reduced glutathione levels and diminished activity of antioxidant enzymes, including superoxide dismutase, glutathione peroxidase, and catalase, have been consistently reported in individuals with ASD [85,86]. This compromised antioxidant capacity renders mitochondria particularly vulnerable to oxidative damage and reinforces redox imbalance.

In ASD, mtDNA-driven immune activation is associated with persistent low-grade inflammation rather than acute tissue injury [87].

This chronic inflammatory state has been observed both in peripheral immune cells and in microglia within the central nervous system, suggesting sustained neuroimmune activation. Such long-term immune signaling may influence synaptic pruning, neuronal connectivity, and brain circuit maturation, thereby contributing to ASD pathophysiology.

### 6.3. Defective Mitophagy, Hypoxia and Neurodevelopmental Vulnerability

Mitochondrial quality control is particularly critical during neurodevelopment, when high energy demands accompany neuronal growth, synaptogenesis, and circuit refinement. Genetic and functional studies implicate impaired mitophagy in ASD pathogenesis, with mutations in genes such as PARK2, WDFY3, AMBRA1, and TSC1/2 disrupting mitochondrial turnover and autophagic processes [88,89,90].

Hyperactivation of mTOR signaling, a common feature in several ASD models, inhibits autophagy and leads to accumulation of dysfunctional mitochondria in neurons [88,91]. This impairs synaptic maturation and network stability, providing a mechanistic link between mitochondrial dysfunction and behavioral phenotypes.

Preciado et al. (2024) reported that prenatal hypoxic risk conditions are associated with increased size of the third ventricle in individuals with ASD, with these changes correlating with sensory hypersensitivity and sleep disturbances [92]. In addition, hypoxia can induce structural brain changes, including reduced brain volume, cortical thinning, and decreased functional connectivity, which contribute to neurodevelopmental impairments [93].

Considering these findings, prenatal hypoxic exposure may be associated with a specific clinical phenotype in ASD [92]. The most metabolically active regions of the fetal and neonatal brain are particularly vulnerable to hypoxic injury and are critical for normal neurological function. Therefore, although hypoxia itself does not determine the pathogenesis of autism, its presence during sensitive periods of brain development may modify the neurodevelopmental trajectory and influence the clinical presentation of symptoms [92].

An increasing number of studies indicate that conditions during pregnancy and birth can significantly influence the manifestation of ASD. Froehlich-Santino et al. (2014) found that respiratory distress associated with hypoxia is strongly linked to an increased risk of developing ASD [94]. Supporting this, elevated serum levels of HIF-1α and apelin have been reported in patients compared to controls.

Prenatal and perinatal hypoxic exposure has been associated with increased ASD risk and altered neurodevelopmental outcomes [94]. Hypoxia during sensitive developmental windows may alter mitochondrial programming, metabolic set points, and immune responses, thereby modifying neurodevelopmental trajectories.

The interaction of hypoxia with underlying mitochondrial and immune instability may also shape the severity of ASD symptoms.

## 7. Mitochondrial Dysfunction as a Common Mechanism in RA, IS, and ASD

Mitochondria play a pivotal role in cellular energy production and regulation of immune responses, making them key players in a variety of pathological conditions. RA, IS, and ASD are distinct diseases with different clinical and sequential courses. Emerging evidence reveals that mitochondrial abnormalities contribute to the pathophysiology of these disorders and highlights a common biological framework (Table 2).

## 8. Strategies to Overcome Mitochondrial Dysfunction

Mitochondrial dysfunction and the accumulation of damaged mitochondria are hallmark features of neurodegenerative disorders. Consequently, enhancing mitophagy—the selective removal of dysfunctional mitochondria—has emerged as a promising therapeutic strategy. To date, several mitophagy modulators with demonstrated neuroprotective effects have been identified, showing potential applicability across diverse pathological conditions [95].

In RA, therapeutic modulation of mitochondrial homeostasis may occur both directly and indirectly. TNF-α inhibitors, widely used in clinical practice, may partially restore mitochondrial function by reducing inflammatory and oxidative stress-mediated mitochondrial damage. In addition, experimental approaches targeting mitophagy regulators or mitochondrial ROS production have demonstrated potential in modulating synovial fibroblast activation and inflammatory cytokine production [50].

Preclinical research indicates that modulating mitochondrial quality control and dynamics through pharmacological or genetic strategies can exert neuroprotective effects in IS [96,97]. The concept of mitochondrial transfer has introduced a new dimension to intercellular communication. Recent evidence suggests that extracellular stress signals can trigger “help-me” responses in damaged mitochondria, prompting neighboring cells to assist and support compromised cells. New therapeutic strategies for hypoxia- and ischemia-related conditions focus on restoring functional mitochondria while removing damaged ones, a process particularly relevant in the central nervous system, where mitochondria are abundant in synapses and dendrites [98]. Stem cells have demonstrated a capacity to preserve mitochondrial function across various preclinical models. They are believed to donate mitochondria to injured cells through tunneling nanotubes, extracellular vesicles, or cell fusion, thereby improving the energetic status of recipient cells [99]. Mitochondrial transplantation has already shown promising results in patients with myocardial ischemia–reperfusion injury, highlighting the potential of stem cell-based approaches for IS treatment. Nevertheless, significant barriers still limit the clinical application of mitochondrial transfer [100].

Currently, there is no curative treatment for ASD and the strategies are focused on symptom management and improvement of adaptive functioning. In recent years, supplements have been used that aim to improve mitochondrial function and bioenergetics, reduce oxidative stress, and support cellular metabolism. Common approaches include dietary supplements with coenzyme Q10, L-carnitine, B vitamins, and antioxidants, which have been shown to improve energy metabolism and, in some cases, behavioral outcomes [38]. Combining these supplements with standard behavioral and supportive therapies allows for a multifaceted, individualized approach that potentially alleviates some of the symptoms of ASD associated with mitochondrial dysfunction.

Among these, NAD^+^ precursors are extensively studied, as intracellular NAD^+^ levels decline with aging and neurodegeneration. NAD^+^ deficiency is closely associated with impaired mitophagy, while supplementation with nicotinamide, nicotinamide mononucleotide, or nicotinamide riboside improves mitochondrial bioenergetics, restores ATP production, reduces ROS accumulation, and alleviates cognitive impairment in Alzheimer’s disease models [101,102,103,104,105].

Urolithin A, a gut microbiota-derived metabolite, induces mitophagy, prevents the accumulation of damaged mitochondria, and preserves mitochondrial respiratory capacity. It improves learning and memory deficits in Alzheimer’s disease models in a PINK1-dependent manner and enhances mitochondrial function in rodents and elderly humans [106,107,108].

Polyamines such as spermidine stimulate autophagy and mitophagy through mTOR inhibition and activation of AMPK and PINK1/PARKIN signaling. Age-related decline in spermidine levels correlates with reduced autophagic capacity, while dietary supplementation promotes longevity, improves cognitive performance, and confers neuroprotection in multiple model organisms and humans [109,110,111,112,113,114].

Additional natural compounds, including tomatidine, anthocyanidins, astragaloside IV, genistein, and p-coumaric acid, modulate autophagy and mitophagy, attenuate oxidative stress, and exert neuroprotective effects in experimental models of neurodegeneration and cerebral ischemia [115,116,117,118,119,120,121].

Pharmacological agents such as rapamycin and metformin similarly induce mitophagy via mTOR and PINK1/PARKIN pathways and demonstrate beneficial effects in models of Alzheimer’s disease, Huntington’s disease, and tau pathology [122,123,124].

Beyond pharmacological modulation, mitochondrial transplantation has recently gained attention as an innovative therapeutic approach. The delivery of functional mitochondria into the central nervous system via intracerebral, intraventricular, or intraarterial routes has shown potential to restore mitochondrial function, particularly following ischemia/reperfusion injury, with transplanted organelles occasionally integrating into host neural cells [125].

Table 3 summarizes the origin and effect of the applied therapeutic agents.

## 9. Conclusions

Mitochondrial dysfunction represents a convergent pathogenic node linking acute ischemic injury, chronic autoimmune inflammation, and neurodevelopmental disorders. Although RA, IS, and ASD differ markedly in clinical presentation and course dynamics, shared disturbances in mitochondrial metabolism, redox balance, innate immune signaling, and quality control underscore the central role of mitochondria in these diseases’ pathogenesis. Targeting mitochondrial function therefore offers promising opportunities for cross-disciplinary therapeutic strategies.

## Figures and Tables

**Table 1:** Major methods for assessment of mitochondrial function.

Method/Parameter	What Is Measured	Biological Material	Advantages	Limitations	Typical Applications
Oxygen consumption (OCR)	Electron transport chain and oxidative phosphorylation activity	Isolated mitochondria, permeabilized or intact cells	High sensitivity; functional, real-time assessment	Requires specialized equipment and careful experimental design	Bioenergetic profiling, metabolic phenotyping
Mitochondrial membrane potential (ΔΨm)	Proton electrochemical gradient	Live cells	Sensitive early marker of dysfunction	Semi-quantitative; influenced by probe conditions	Apoptosis, cellular stress
ATP production	Cellular or mitochondrial energy output	Cells, tissues, mitochondria	Direct measure of energetic function	Does not distinguish between mitochondrial and glycolytic sources	Metabolic and pharmacological studies
Mitochondrial ROS production	Oxidative stress levels	Live cells, mitochondria	Relevant to pathophysiology and signaling	Limited probe specificity and potential for signal artifacts due to the highly reactive and transient nature of ROS	Aging, neurodegeneration, toxicity
ETC complex activity assays	Activity of complexes I–IV	Tissues, isolated mitochondria	Enables localization of specific defects	Performed under in vitro conditions and do not reflect mitochondrial dynamics or regulation in intact cells	Mitochondrial disease diagnostics
Mitochondrial mass	Relative mitochondrial content	Cells, tissues	Simple quantitative assessment	Does not directly reflect mitochondrial functional status or bioenergetic capacity	Biogenesis and adaptation studies
Morphology and dynamics	Fission, fusion, network organization	Cells, tissues	Links structure to function	Provides indirect functional insight and typically requires complementary functional assays for interpretation	Stress responses, metabolic remodeling

**Table 2:** Shared pathophysiological mechanisms associated with mitochondrial dysfunction.

Mechanism	RA	IS	ASD
Metabolism	Glycolytic shift in immune cells	Acute OXPHOS failure; glycolytic compensation	Reduced metabolic flexibility; glycolytic bias
ROS	Chronic ROS-sustained inflammation	Excess ROS during ischemia–reperfusion	Persistent oxidative stress
mtDNA/innate immunity	Chronic sterile inflammation	Acute inflammatory amplification	Low-grade chronic immune activation
Mitophagy	Defective mitochondrial clearance	Protective in early stages	Impaired during neurodevelopment
Hypoxia	Chronic synovial hypoxia	Acute ischemia with reperfusion injury	Developmental hypoxic brain vulnerability
Cellular outcome	Persistence of chronic inflammation, tissue and joint destruction	Neuronal death and secondary brain injury	Functional dysregulation of the central nervous system

**Table 3:** Main mitophagy modulators used in current therapeutic strategies.

Mitophagy Modulator	Origin	Primary Target	Target and Effects
NAD^+^ precursors	Human-derived	SIRT1/SIRT3 activation; PGC-1α signaling; NAD^+^-dependent deacetylase pathways; mitophagy regulation via PINK1/PARKIN axis.	Improve brain bioenergetics with preserved functionality of mitochondria and the autophagy system and restore ATP levels and attenuate the accumulation of ROS in Aβ oligomer-treated hippocampal tissue.
Urolithin A	Microflora-derived	PINK1/PARKIN-dependent mitophagy pathway; mitochondrial quality control signaling; AMPK activation.	Prevents the accumulation of damaged mitochondria, maintains mitochondrial respiratory capacity, and extends healthspan and lifespan through the induction of mitophagy.
Spermidine	Plant-derived	mTORC1 inhibition; AMPK activation; EP300 inhibition; autophagy–mitophagy regulatory axis.	Enhances mitophagy through mTOR inhibition and AMP-activated protein kinase and PINK1/PARKIN activation.
Tomatidine	Plant-derived	PGC-1α signaling; ATF4 pathway; mitochondrial biogenesis regulators; mitophagy-related pathways.	Induces mitophagy and promotes mitochondrial biogenesis.
Rapamycin	Bacteria-derived	mTORC1 (mechanistic target of rapamycin complex 1); ULK1 autophagy initiation complex.	mTOR inhibitor which enhances the level of LC3 II, PARKIN, and BECLIN-1 in the hippocampus of AD mice.
Metformin	Plant-derived	AMPK activation; mitochondrial complex I (indirect inhibition); PINK1/PARKIN mitophagy signaling.	Induces mitophagy by up-regulation of the PINK1/PARKIN pathway.
Anthocyanidin	Plant-derived	Nrf2/ARE antioxidant pathway; mitochondrial ROS signaling; AMPK.	Activates autophagy, decreases oxidative stress and protects glial cells subjected to oxygen-glucose deprivation.
Astragaloside IV	Plant-derived	PI3K/Akt signaling; AMPK pathway; autophagy/mitophagy regulatory proteins (LC3, Beclin-1).	Plays neuroprotective role and promotes autophagy.
Curcumin	Plant-derived	Nrf2 pathway; NF-κB inhibition; AMPK activation; mitochondrial apoptotic signaling (Bcl-2/Bax axis).	Has neuroprotective effects and inhibits autophagy and apoptosis.
Glycyrrhizic acid	Plant-derived	HMGB1 inhibition; autophagy-related signaling (Beclin-1, LC3); anti-inflammatory mitochondrial stress pathways.	Induces autophagy and upregulates LC3B II/I conversion, BECLIN 1 expression, and autophagy in neuroblastoma cells.
Genistein	Plant-derived	mTOR inhibition; PI3K/Akt modulation; lysosomal biogenesis (TFEB-related pathways); autophagy–mitophagy axis.	Induces mitophagy by inactivating mTOR signaling and enhances lysosomal activities.
P-coumaric acid	Plant-derived	Nrf2 antioxidant signaling; ROS-sensitive mitochondrial pathways; autophagy-related signaling cascades.	Causes growth arrest by activating autophagy.

## Data Availability

No new data were created or analyzed in this study. Data sharing is not applicable to this article.

## References

[1] Andrieux P., Chevillard C., Cunha-Neto E., Nunes J.P.S. (2021). Mitochondria as a Cellular Hub in Infection and Inflammation. Int. J. Mol. Sci..

[2] Guan S., Zhao L., Peng R. (2022). Mitochondrial Respiratory Chain Supercomplexes: From Structure to Function. Int. J. Mol. Sci..

[3] Protasoni M., Zeviani M. (2021). Mitochondrial Structure and Bioenergetics in Normal and Disease Conditions. Int. J. Mol. Sci..

[4] Guo R., Gu J., Zong S., Wu M., Yang M. (2018). Structure and mechanism of mitochondrial electron transport chain. Biomed. J..

[5] Nolfi-Donegan D., Braganza A., Shiva S. (2020). Mitochondrial electron transport chain: Oxidative phosphorylation, oxidant production, and methods of measurement. Redox Biol..

[6] Kukulski F., Ben Yebdri F., Lecka J., Kauffenstein G., Levesque S.A., Martin-Satue M., Sevigny J. (2009). Extracellular ATP and P2 receptors are required for IL-8 to induce neutrophil migration. Cytokine.

[7] Gombault A., Baron L., Couillin I. (2012). ATP release and purinergic signaling in NLRP3 inflammasome activation. Front. Immunol..

[8] Mortaz E., Adcock I.M., Shafei H., Masjedi M.R., Folkerts G. (2012). Role of P2X7 Receptors in Release of IL-1beta: A Possible Mediator of Pulmonary Inflammation. Tanaffos.

[9] Moreno-Lastres D., Fontanesi F., Garcia-Consuegra I., Martin M.A., Arenas J., Barrientos A., Ugalde C. (2012). Mitochondrial complex I plays an essential role in human respirasome assembly. Cell Metab..

[10] Tang J.X., Thompson K., Taylor R.W., Olahova M. (2020). Mitochondrial OXPHOS Biogenesis: Co-Regulation of Protein Synthesis, Import, and Assembly Pathways. Int. J. Mol. Sci..

[11] Greene J., Segaran A., Lord S. (2022). Targeting OXPHOS and the electron transport chain in cancer; Molecular and therapeutic implications. Semin. Cancer Biol..

[12] Pereira S.P., Tavares L.C. (2025). Beyond Powerhouses: Roles of Mitochondria, from Development to Therapeutic Potential. Biology.

[13] Valera-Alberni M., Canto C. (2018). Mitochondrial stress management: A dynamic journey. Cell Stress..

[14] Kim M.E., Lim Y., Lee J.S. (2025). Mitochondrial Dysfunction and Metabolic Reprogramming in Chronic Inflammatory Diseases: Molecular Insights and Therapeutic Opportunities. Curr. Issues Mol. Biol..

[15] Zong Y., Li H., Liao P., Chen L., Pan Y., Zheng Y., Zhang C., Liu D., Zheng M., Gao J. (2024). Mitochondrial dysfunction: Mechanisms and advances in therapy. Signal Transduct. Target. Ther..

[16] Taherzadeh-Fard E., Saft C., Akkad D.A., Wieczorek S., Haghikia A., Chan A., Epplen J.T., Arning L. (2011). PGC-1alpha downstream transcription factors NRF-1 and TFAM are genetic modifiers of Huntington disease. Mol. Neurodegener..

[17] Green A., Hossain T., Eckmann D.M. (2022). Mitochondrial dynamics involves molecular and mechanical events in motility, fusion and fission. Front. Cell Dev. Biol..

[18] Tait S.W., Green D.R. (2013). Mitochondrial regulation of cell death. Cold Spring Harb. Perspect. Biol..

[19] Lin R.Z., Im G.B., Luo A.C., Zhu Y., Hong X., Neumeyer J., Tang H.W., Perrimon N., Melero-Martin J.M. (2024). Mitochondrial transfer mediates endothelial cell engraftment through mitophagy. Nature.

[20] Hamanaka R.B., Chandel N.S. (2010). Mitochondrial reactive oxygen species regulate cellular signaling and dictate biological outcomes. Trends Biochem. Sci..

[21] Olsen R.K., Cornelius N., Gregersen N. (2015). Redox signalling and mitochondrial stress responses; lessons from inborn errors of metabolism. J. Inherit. Metab. Dis..

[22] Kowalczyk P., Sulejczak D., Kleczkowska P., Bukowska-Osko I., Kucia M., Popiel M., Wietrak E., Kramkowski K., Wrzosek K., Kaczynska K. (2021). Mitochondrial Oxidative Stress—A Causative Factor and Therapeutic Target in Many Diseases. Int. J. Mol. Sci..

[23] Nakahira K., Hisata S., Choi A.M. (2015). The Roles of Mitochondrial Damage—Associated Molecular Patterns in Diseases. Antioxid. Redox Signal..

[24] Zhou Z., Ou-Yang C., Chen Q., Ren Z., Guo X., Lei M., Liu C., Yang X. (2023). Trafficking and effect of released DNA on cGAS-STING signaling pathway and cardiovascular disease. Front. Immunol..

[25] Alvarez S., Vanasco V., Adan Arean J.S., Magnani N., Evelson P. (2024). Mitochondrial Mechanisms in Immunity and Inflammatory Conditions: Beyond Energy Management. Antioxid. Redox Signal..

[26] Trinchese G., Cimmino F., Catapano A., Cavaliere G., Mollica M.P. (2024). Mitochondria: The gatekeepers between metabolism and immunity. Front. Immunol..

[27] Rangaraju V., Lewis T.L., Hirabayashi Y., Bergami M., Motori E., Cartoni R., Kwon S.K., Courchet J. (2019). Pleiotropic Mitochondria: The Influence of Mitochondria on Neuronal Development and Disease. J. Neurosci..

[28] Nesci S., Lenaz G. (2022). Impaired Mitochondrial Bioenergetics under Pathological Conditions. Life.

[29] Ma C., Wang J., Hong F., Yang S. (2022). Mitochondrial Dysfunction in Rheumatoid Arthritis. Biomolecules.

[30] Sharma E., Fotooh Abadi L., Kombe Kombe J.A., Kandala M., Parker J., Winicki N., Kelesidis T. (2025). Overview of methods that determine mitochondrial function in human disease. Metabolism.

[31] Tovar-Ferrero O., Rubio J., Zorzano A., Martinez-Corrales G., Liesa M. (2025). Measuring mitochondrial membrane potential. EMBO J..

[32] Yu L., Fink B.D., Sivitz W.I. (2015). Simultaneous quantification of mitochondrial ATP and ROS production. Methods Mol. Biol..

[33] Robinson K.M., Janes M.S., Pehar M., Monette J.S., Ross M.F., Hagen T.M., Murphy M.P., Beckman J.S. (2006). Selective fluorescent imaging of superoxide in vivo using ethidium-based probes. Proc. Natl. Acad. Sci. USA.

[34] Spinazzi M., Casarin A., Pertegato V., Salviati L., Angelini C. (2012). Assessment of mitochondrial respiratory chain enzymatic activities on tissues and cultured cells. Nat. Protoc..

[35] Yin Y., Shen H. (2022). Common methods in mitochondrial research (Review). Int. J. Mol. Med..

[36] Chen T., Zhou Z., Liu Y., Xu J., Zhu C., Sun R., Hu H., Liu Y., Dai L., Holmdahl R. (2024). Neutrophils with low production of reactive oxygen species are activated during immune priming and promote development of arthritis. Redox Biol..

[37] An H., Zhou B., Ji X. (2021). Mitochondrial quality control in acute ischemic stroke. J. Cereb. Blood Flow. Metab..

[38] Frye R.E. (2020). Mitochondrial Dysfunction in Autism Spectrum Disorder: Unique Abnormalities and Targeted Treatments. Semin. Pediatr. Neurol..

[39] Mihaylova V., Kazakova M., Batalov Z., Karalilova R., Batalov A., Sarafian V. (2024). JAK inhibitors improve ATP production and mitochondrial function in rheumatoid arthritis: A pilot study. Rheumatol. Int..

[40] Bustamante M.F., Garcia-Carbonell R., Whisenant K.D., Guma M. (2017). Fibroblast-like synoviocyte metabolism in the pathogenesis of rheumatoid arthritis. Arthritis Res. Ther..

[41] Otero M., Goldring M.B. (2007). Cells of the synovium in rheumatoid arthritis. Chondrocytes. Arthritis Res. Ther..

[42] Di Matteo A., Bathon J.M., Emery P. (2023). Rheumatoid arthritis. Lancet.

[43] Vander Heiden M.G., Cantley L.C., Thompson C.B. (2009). Understanding the Warburg effect: The metabolic requirements of cell proliferation. Science.

[44] Shi Y., Zhang H., Miao C. (2025). Metabolic reprogram and T cell differentiation in inflammation: Current evidence and future perspectives. Cell Death Discov..

[45] Weyand C.M., Zeisbrich M., Goronzy J.J. (2017). Metabolic signatures of T-cells and macrophages in rheumatoid arthritis. Curr. Opin. Immunol..

[46] Algieri C., Nesci S., Oppedisano F. (2025). Mitochondrial dysfunction acts as a modulator of the immunometabolic route for activating the cytosolic DNA sensor pathway in triggering innate immunosurveillance. J. Transl. Med..

[47] You S., Koh J.H., Leng L., Kim W.U., Bucala R. (2018). The Tumor-Like Phenotype of Rheumatoid Synovium: Molecular Profiling and Prospects for Precision Medicine. Arthritis Rheumatol..

[48] Liu S., Liu J., Wang Y., Deng F., Deng Z. (2025). Oxidative Stress: Signaling Pathways, Biological Functions, and Disease. MedComm (2020).

[49] Jing W., Liu C., Su C., Liu L., Chen P., Li X., Zhang X., Yuan B., Wang H., Du X. (2023). Role of reactive oxygen species and mitochondrial damage in rheumatoid arthritis and targeted drugs. Front. Immunol..

[50] Gong X., Yang S.Y., Wang Z.Y., Tang M. (2024). The role of hypoxic microenvironment in autoimmune diseases. Front. Immunol..

[51] Bouhamida E., Morciano G., Perrone M., Kahsay A.E., Della Sala M., Wieckowski M.R., Fiorica F., Pinton P., Giorgi C., Patergnani S. (2022). The Interplay of Hypoxia Signaling on Mitochondrial Dysfunction and Inflammation in Cardiovascular Diseases and Cancer: From Molecular Mechanisms to Therapeutic Approaches. Biology.

[52] Zhong W., Rao Z., Xu J., Sun Y., Hu H., Wang P., Xia Y., Pan X., Tang W., Chen Z. (2022). Defective mitophagy in aged macrophages promotes mitochondrial DNA cytosolic leakage to activate STING signaling during liver sterile inflammation. Aging Cell.

[53] Iaconis A., Molinari F., Fusco R., Di Paola R. (2025). Mitochondria as a Disease-Relevant Organelle in Rheumatoid Arthritis: A Key Breakout in Fight Against the Disease. Biomedicines.

[54] Wang Q., Sun Y., Li T.Y., Auwerx J. (2026). Mitophagy in the pathogenesis and management of disease. Cell Res..

[55] Nam J.H., Lee J.H., Choi H.J., Choi S.Y., Noh K.E., Jung N.C., Song J.Y., Choi J., Seo H.G., Jung S.Y. (2022). TNF-alpha Induces Mitophagy in Rheumatoid Arthritis Synovial Fibroblasts, and Mitophagy Inhibition Alleviates Synovitis in Collagen Antibody-Induced Arthritis. Int. J. Mol. Sci..

[56] Riitano G., Recalchi S., Capozzi A., Manganelli V., Misasi R., Garofalo T., Sorice M., Longo A. (2023). The Role of Autophagy as a Trigger of Post-Translational Modifications of Proteins and Extracellular Vesicles in the Pathogenesis of Rheumatoid Arthritis. Int. J. Mol. Sci..

[57] Schneider J., Berndt N., Papageorgiou I.E., Maurer J., Bulik S., Both M., Draguhn A., Holzhutter H.G., Kann O. (2019). Local oxygen homeostasis during various neuronal network activity states in the mouse hippocampus. J. Cereb. Blood Flow. Metab..

[58] Liang J., Han R., Zhou B. (2021). Metabolic Reprogramming: Strategy for Ischemic Stroke Treatment by Ischemic Preconditioning. Biology.

[59] Wang C., Li L. (2023). The critical role of KLF4 in regulating the activation of A1/A2 reactive astrocytes following ischemic stroke. J. Neuroinflammation.

[60] Zhang H., Zheng Q., Guo T., Zhang S., Zheng S., Wang R., Deng Q., Yang G., Zhang S., Tang L. (2024). Metabolic reprogramming in astrocytes results in neuronal dysfunction in intellectual disability. Mol. Psychiatry.

[61] Kuroda S., Katsura K.I., Tsuchidate R., Siesjo B.K. (1996). Secondary bioenergetic failure after transient focal ischaemia is due to mitochondrial injury. Acta Physiol. Scand..

[62] Chouchani E.T., Pell V.R., Gaude E., Aksentijevic D., Sundier S.Y., Robb E.L., Logan A., Nadtochiy S.M., Ord E.N.J., Smith A.C. (2014). Ischaemic accumulation of succinate controls reperfusion injury through mitochondrial ROS. Nature.

[63] Shaafi S., Hadisi F., Mahmoudinezhad M., Razmi H., Nejadghaderi S.A., Khalili M. (2021). The significance of the oxidative stress markers in the one-year prognosis of patients with acute ischemic stroke: A case-control study. BMC Neurol..

[64] Kunz A., Park L., Abe T., Gallo E.F., Anrather J., Zhou P., Iadecola C. (2007). Neurovascular protection by ischemic tolerance: Role of nitric oxide and reactive oxygen species. J. Neurosci..

[65] He Z., Ning N., Zhou Q., Khoshnam S.E., Farzaneh M. (2020). Mitochondria as a therapeutic target for ischemic stroke. Free Radic. Biol. Med..

[66] Walko T.D., Bola R.A., Hong J.D., Au A.K., Bell M.J., Kochanek P.M., Clark R.S., Aneja R.K. (2014). Cerebrospinal fluid mitochondrial DNA: A novel DAMP in pediatric traumatic brain injury. Shock..

[67] He Q., Li Z., Meng C., Wu J., Zhao Y., Zhao J. (2019). Parkin-Dependent Mitophagy is Required for the Inhibition of ATF4 on NLRP3 Inflammasome Activation in Cerebral Ischemia-Reperfusion Injury in Rats. Cells.

[68] Shao Z., Dou S., Zhu J., Wang H., Xu D., Wang C., Cheng B., Bai B. (2020). The Role of Mitophagy in Ischemic Stroke. Front. Neurol..

[69] Domazou A.S., Gebicka L., Didik J., Gebicki J.L., van der Meijden B., Koppenol W.H. (2014). The kinetics of the reaction of nitrogen dioxide with iron(II)- and iron(III) cytochrome c. Free Radic. Biol. Med..

[70] Mo Y., Sun Y.Y., Liu K.Y. (2020). Autophagy and inflammation in ischemic stroke. Neural Regen. Res..

[71] Hussain T., Tan B., Yin Y., Blachier F., Tossou M.C., Rahu N. (2016). Oxidative Stress and Inflammation: What Polyphenols Can Do for Us?. Oxid. Med. Cell Longev..

[72] Hughes H.K., Moreno R.J., Ashwood P. (2023). Innate immune dysfunction and neuroinflammation in autism spectrum disorder (ASD). Brain Behav. Immun..

[73] Haas R.H., Parikh S., Falk M.J., Saneto R.P., Wolf N.I., Darin N., Cohen B.H. (2007). Mitochondrial disease: A practical approach for primary care physicians. Pediatrics.

[74] Morris G., Gevezova M., Sarafian V., Maes M. (2022). Redox regulation of the immune response. Cell Mol. Immunol..

[75] Oliveira G., Ataide A., Marques C., Miguel T.S., Coutinho A.M., Mota-Vieira L., Goncalves E., Lopes N.M., Rodrigues V., Carmona da Mota H. (2007). Epidemiology of autism spectrum disorder in Portugal: Prevalence, clinical characterization, and medical conditions. Dev. Med. Child. Neurol..

[76] Oya M., Matsuoka K., Kubota M., Fujino J., Tei S., Takahata K., Tagai K., Yamamoto Y., Shimada H., Seki C. (2023). Increased glutamate and glutamine levels and their relationship to astrocytes and dopaminergic transmissions in the brains of adults with autism. Sci. Rep..

[77] Shimmura C., Suda S., Tsuchiya K.J., Hashimoto K., Ohno K., Matsuzaki H., Iwata K., Matsumoto K., Wakuda T., Kameno Y. (2011). Alteration of plasma glutamate and glutamine levels in children with high-functioning autism. PLoS ONE.

[78] Frye R.E., Melnyk S., Macfabe D.F. (2013). Unique acyl-carnitine profiles are potential biomarkers for acquired mitochondrial disease in autism spectrum disorder. Transl. Psychiatry.

[79] Gevezova M., Minchev D., Pacheva I., Sbirkov Y., Yordanova R., Timova E., Kotetarov V., Ivanov I., Sarafian V. (2021). Cellular Bioenergetic and Metabolic Changes in Patients with Autism Spectrum Disorder. Curr. Top. Med. Chem..

[80] Hu T., Liu C.H., Lei M., Zeng Q., Li L., Tang H., Zhang N. (2024). Metabolic regulation of the immune system in health and diseases: Mechanisms and interventions. Signal Transduct. Target. Ther..

[81] Naviaux R.K. (2023). Mitochondrial and metabolic features of salugenesis and the healing cycle. Mitochondrion.

[82] Naviaux R.K. (2018). Antipurinergic therapy for autism—An in-depth review. Mitochondrion.

[83] Rossignol D.A., Frye R.E. (2012). Mitochondrial dysfunction in autism spectrum disorders: A systematic review and meta-analysis. Mol. Psychiatry.

[84] Rossignol D.A., Frye R.E. (2012). A review of research trends in physiological abnormalities in autism spectrum disorders: Immune dysregulation, inflammation, oxidative stress, mitochondrial dysfunction and environmental toxicant exposures. Mol. Psychiatry.

[85] Melnyk S., Fuchs G.J., Schulz E., Lopez M., Kahler S.G., Fussell J.J., Bellando J., Pavliv O., Rose S., Seidel L. (2012). Metabolic imbalance associated with methylation dysregulation and oxidative damage in children with autism. J. Autism Dev. Disord..

[86] Frye R.E., Delatorre R., Taylor H., Slattery J., Melnyk S., Chowdhury N., James S.J. (2013). Redox metabolism abnormalities in autistic children associated with mitochondrial disease. Transl. Psychiatry.

[87] Qu W., Yan G., Du Y., Zhou X., Huang C., Li B., Zhou J., Li Q. (2025). Crosstalk Between Mitochondrial DNA and Immune Response: Focus on Autism Spectrum Disorder. Mol. Neurobiol..

[88] Tang G., Gudsnuk K., Kuo S.H., Cotrina M.L., Rosoklija G., Sosunov A., Sonders M.S., Kanter E., Castagna C., Yamamoto A. (2014). Loss of mTOR-dependent macroautophagy causes autistic-like synaptic pruning deficits. Neuron.

[89] Napoli E., Song G., Panoutsopoulos A., Riyadh M.A., Kaushik G., Halmai J., Levenson R., Zarbalis K.S., Giulivi C. (2018). Beyond autophagy: A novel role for autism-linked Wdfy3 in brain mitophagy. Sci. Rep..

[90] Dalla Vecchia E., Mortimer N., Palladino V.S., Kittel-Schneider S., Lesch K.P., Reif A., Schenck A., Norton W.H.J. (2019). Cross-species models of attention-deficit/hyperactivity disorder and autism spectrum disorder: Lessons from CNTNAP2, ADGRL3, and PARK2. Psychiatr. Genet..

[91] Goorden S.M., van Woerden G.M., van der Weerd L., Cheadle J.P., Elgersma Y. (2007). Cognitive deficits in Tsc1+/− mice in the absence of cerebral lesions and seizures. Ann. Neurol..

[92] Preciado C., Baida M., Li Y., Li Y., Demopoulos C. (2024). Prenatal exposure to hypoxic risk conditions in autistic and neurotypical youth: Associated ventricular differences, sleep disturbance, and sensory processing. Autism Res..

[93] Miller S.L., Huppi P.S., Mallard C. (2016). The consequences of fetal growth restriction on brain structure and neurodevelopmental outcome. J. Physiol..

[94] Froehlich-Santino W., Londono Tobon A., Cleveland S., Torres A., Phillips J., Cohen B., Torigoe T., Miller J., Fedele A., Collins J. (2014). Prenatal and perinatal risk factors in a twin study of autism spectrum disorders. J. Psychiatr. Res..

[95] Fang E.F. (2019). Mitophagy and NAD^+^ inhibit Alzheimer disease. Autophagy.

[96] Tucker D., Lu Y., Zhang Q. (2018). From Mitochondrial Function to Neuroprotection—An Emerging Role for Methylene Blue. Mol. Neurobiol..

[97] Narayanan S.V., Dave K.R., Saul I., Perez-Pinzon M.A. (2015). Resveratrol Preconditioning Protects Against Cerebral Ischemic Injury via Nuclear Erythroid 2-Related Factor 2. Stroke.

[98] Egawa N., Lok J., Washida K., Arai K. (2017). Mechanisms of Axonal Damage and Repair after Central Nervous System Injury. Transl. Stroke Res..

[99] Sarmah D., Kaur H., Saraf J., Vats K., Pravalika K., Wanve M., Kalia K., Borah A., Kumar A., Wang X. (2018). Mitochondrial Dysfunction in Stroke: Implications of Stem Cell Therapy. Transl. Stroke Res..

[100] Emani S.M., Piekarski B.L., Harrild D., Del Nido P.J., McCully J.D. (2017). Autologous mitochondrial transplantation for dysfunction after ischemia-reperfusion injury. J. Thorac. Cardiovasc. Surg..

[101] Fang E.F., Scheibye-Knudsen M., Brace L.E., Kassahun H., SenGupta T., Nilsen H., Mitchell J.R., Croteau D.L., Bohr V.A. (2014). Defective mitophagy in XPA via PARP-1 hyperactivation and NAD^+^/SIRT1 reduction. Cell.

[102] Fang E.F., Kassahun H., Croteau D.L., Scheibye-Knudsen M., Marosi K., Lu H., Shamanna R.A., Kalyanasundaram S., Bollineni R.C., Wilson M.A. (2016). NAD^+^ Replenishment Improves Lifespan and Healthspan in Ataxia Telangiectasia Models via Mitophagy and DNA Repair. Cell Metab..

[103] Lou G., Palikaras K., Lautrup S., Scheibye-Knudsen M., Tavernarakis N., Fang E.F. (2020). Mitophagy and Neuroprotection. Trends Mol. Med..

[104] Liu D., Pitta M., Jiang H., Lee J.H., Zhang G., Chen X., Kawamoto E.M., Mattson M.P. (2013). Nicotinamide forestalls pathology and cognitive decline in Alzheimer mice: Evidence for improved neuronal bioenergetics and autophagy procession. Neurobiol. Aging.

[105] Wang X., Tang L., Gao L., Yang Y., Cao D., Li Y. (2016). Myopia and diabetic retinopathy: A systematic review and meta-analysis. Diabetes Res. Clin. Pract..

[106] Ryu D., Mouchiroud L., Andreux P.A., Katsyuba E., Moullan N., Nicolet-Dit-Felix A.A., Williams E.G., Jha P., Lo Sasso G., Huzard D. (2016). Urolithin A induces mitophagy and prolongs lifespan in *C. elegans* and increases muscle function in rodents. Nat. Med..

[107] Andreux P.A., Blanco-Bose W., Ryu D., Burdet F., Ibberson M., Aebischer P., Auwerx J., Singh A., Rinsch C. (2019). The mitophagy activator urolithin A is safe and induces a molecular signature of improved mitochondrial and cellular health in humans. Nat. Metab..

[108] Fang E.F., Hou Y., Palikaras K., Adriaanse B.A., Kerr J.S., Yang B., Lautrup S., Hasan-Olive M.M., Caponio D., Dan X. (2019). Mitophagy inhibits amyloid-beta and tau pathology and reverses cognitive deficits in models of Alzheimer’s disease. Nat. Neurosci..

[109] Madeo F., Eisenberg T., Pietrocola F., Kroemer G. (2018). Spermidine in health and disease. Science.

[110] Fan J., Yang X., Li J., Shu Z., Dai J., Liu X., Li B., Jia S., Kou X., Yang Y. (2017). Spermidine coupled with exercise rescues skeletal muscle atrophy from D-gal-induced aging rats through enhanced autophagy and reduced apoptosis via AMPK-FOXO3a signal pathway. Oncotarget.

[111] Gupta V.K., Scheunemann L., Eisenberg T., Mertel S., Bhukel A., Koemans T.S., Kramer J.M., Liu K.S., Schroeder S., Stunnenberg H.G. (2013). Restoring polyamines protects from age-induced memory impairment in an autophagy-dependent manner. Nat. Neurosci..

[112] Eisenberg T., Knauer H., Schauer A., Buttner S., Ruckenstuhl C., Carmona-Gutierrez D., Ring J., Schroeder S., Magnes C., Antonacci L. (2009). Induction of autophagy by spermidine promotes longevity. Nat. Cell Biol..

[113] Eisenberg T., Abdellatif M., Schroeder S., Primessnig U., Stekovic S., Pendl T., Harger A., Schipke J., Zimmermann A., Schmidt A. (2016). Cardioprotection and lifespan extension by the natural polyamine spermidine. Nat. Med..

[114] Yang X., Zhang M., Dai Y., Sun Y., Aman Y., Xu Y., Yu P., Zheng Y., Yang J., Zhu X. (2020). Spermidine inhibits neurodegeneration and delays aging via the PINK1-PDR1-dependent mitophagy pathway in *C. elegans*. Aging.

[115] Fang E.F., Waltz T.B., Kassahun H., Lu Q., Kerr J.S., Morevati M., Fivenson E.M., Wollman B.N., Marosi K., Wilson M.A. (2017). Tomatidine enhances lifespan and healthspan in *C. elegans* through mitophagy induction via the SKN-1/Nrf2 pathway. Sci. Rep..

[116] Chiu F.L., Lin J.K. (2008). Tomatidine inhibits iNOS and COX-2 through suppression of NF-kappaB and JNK pathways in LPS-stimulated mouse macrophages. FEBS Lett..

[117] Huang S.L., He H.B., Zou K., Bai C.H., Xue Y.H., Wang J.Z., Chen J.F. (2014). Protective effect of tomatine against hydrogen peroxide-induced neurotoxicity in neuroblastoma (SH-SY5Y) cells. J. Pharm. Pharmacol..

[118] Ravikumar B., Vacher C., Berger Z., Davies J.E., Luo S., Oroz L.G., Scaravilli F., Easton D.F., Duden R., O’Kane C.J. (2004). Inhibition of mTOR induces autophagy and reduces toxicity of polyglutamine expansions in fly and mouse models of Huntington disease. Nat. Genet..

[119] Wang C., Yang Y., Zhang Y., Liu J., Yao Z., Zhang C. (2019). Protective effects of metformin against osteoarthritis through upregulation of SIRT3-mediated PINK1/Parkin-dependent mitophagy in primary chondrocytes. Biosci. Trends.

[120] Lin B.W., Gong C.C., Song H.F., Cui Y.Y. (2017). Effects of anthocyanins on the prevention and treatment of cancer. Br. J. Pharmacol..

[121] Kim Y.K., Yoon H.H., Lee Y.D., Youn D.Y., Ha T.J., Kim H.S., Lee J.H. (2012). Anthocyanin Extracts from Black Soybean (*Glycine max* L.) Protect Human Glial Cells Against Oxygen-Glucose Deprivation by Promoting Autophagy. Biomol. Ther..

[122] Yang C., Mo Y., Xu E., Wen H., Wei R., Li S., Zheng J., Li W., Le B., Chen Y. (2019). Astragaloside IV ameliorates motor deficits and dopaminergic neuron degeneration via inhibiting neuroinflammation and oxidative stress in a Parkinson’s disease mouse model. Int. Immunopharmacol..

[123] Zhang Y., Zhang Y., Jin X.F., Zhou X.H., Dong X.H., Yu W.T., Gao W.J. (2019). The Role of Astragaloside IV against Cerebral Ischemia/Reperfusion Injury: Suppression of Apoptosis via Promotion of P62-LC3-Autophagy. Molecules.

[124] Ahsan A., Liu M., Zheng Y., Yan W., Pan L., Li Y., Ma S., Zhang X., Cao M., Wu Z. (2021). Natural compounds modulate the autophagy with potential implication of stroke. Acta Pharm. Sin. B.

[125] Norat P., Soldozy S., Sokolowski J.D., Gorick C.M., Kumar J.S., Chae Y., Yagmurlu K., Prada F., Walker M., Levitt M.R. (2020). Mitochondrial dysfunction in neurological disorders: Exploring mitochondrial transplantation. npj Regen. Med..

